# Multifunctional Starch-Based Composite Films Embedded with Carbon Dots for pH-Responsive Sensing and Monitoring of Food Freshness

**DOI:** 10.3390/molecules30224421

**Published:** 2025-11-15

**Authors:** Xiaoxu Zhang, Chengguo Liu, Xiaoqin Yang, Qian Jiang, Can Liu, Ping Zhao

**Affiliations:** 1 National Joint Engineering Research Center for Highly-Efficient Utilization Technology of Forestry Resources, Southwest Forestry University, Kunming 650224, China; 18183867795@163.com (X.Z.); liuchengguo@njfu.edu.cn (C.L.); yangxiaoqin@swfu.edu.cn (X.Y.); jqian202304@163.com (Q.J.); liucan@swfu.edu.cn (C.L.); 2 College of Chemical Engineering, Jiangsu Co-Innovation Center of Efficient Processing and Utilization of Forest Resources, Jiangsu Key Lab for the Chemistry and Utilization of Agricultural and Forest Biomass, Nanjing Forestry University, Nanjing 210037, China

**Keywords:** carbon dots, starch-based film, pH-responsive, electrochemical sensor, intelligent packaging, rapid food detection

## Abstract

This study presents the development of a starch-based smart film enhanced with carbon dots (CDs), which exhibits high pH sensitivity for the rapid assessment of liquid food quality. CDs were synthesized from ellagic acid and urea as precursors and incorporated into amylopectin (CA) and whole-component starch (CS) matrices, yielding a range of starch-based/CDs composite films. Structural analyses indicated that hydrogen bonding interactions between the CDs and starch molecules not only provided a nano-plasticizing effect-significantly improving film flexibility, as evidenced by a 29.6-fold increase in the elongation at break of the CS/CDs-2 film—but also slightly enhanced the crystallinity by acting as a nucleating agent. The incorporation of CDs markedly improved the electrochemical properties of the films, reducing the electrical impedance by several orders of magnitude and imparting intense blue fluorescence. The fluorescence intensity and current response of the composite film were found to be highly sensitive to pH variations. A strong linear positive correlation (*R*^2^ > 0.97) was observed between the current output and pH over the range of 2 to 7. In a simulated orange juice spoilage test, the film effectively distinguished between fresh (pH = 3.38) and spoiled (pH = 2.68) samples based on current signal differences, demonstrating its practical utility. This work offers a novel approach for designing low-cost, biodegradable sensing materials for smart packaging, with promising potential in real-time food freshness monitoring.

## 1. Introduction

Food safety and quality monitoring represent critical global challenges, driving research efforts toward intelligent packaging technologies that enable real-time, rapid, and non-destructive assessment of food conditions [1,2]. While conventional analytical techniques—such as high-performance liquid chromatography [3,4] and gas chromatography–mass spectrometry [5,6], Although this method is accuracy, they are often hampered by inherent drawbacks including time-consuming procedures, operational complexity, and destructive sampling. These limitations restrict their applicability for real-time, in situ food quality monitoring in modern supply chains. In this context, the integration of sensing functions into intelligent packaging materials facilitates the real-time monitoring and visual assessment of food quality [7,8].

Among various indicators of food spoilage, pH represents a critical parameter. The spoilage of many liquid foods, such as fruit juices and dairy products, often involves microbial activity that generates organic acids, resulting in a pronounced decrease in pH [9]. Consequently, the development of sensing materials with high sensitivity to pH variation is essential. An ideal sensing material should combine excellent detection performance with environmental sustainability. Biodegradable films derived from natural polymers, including starch and chitosan, are considered promising alternatives to conventional plastics, owing to their renewability and biodegradability [10,11]. However, their inherent lack of functionality—such as electrical conductivity and fluorescence—limits their practical application in intelligent packaging systems [12]. Thus, the functional modification of natural polymers remains a key research challenge.

Incorporating functional nanomaterials into natural polymer matrices presents a viable strategy to address these limitations. Commonly employed nanofillers range from metal nanoparticles and metal oxides to various carbon-based nanomaterials, including the emerging carbon dots (CDs) [13,14,15,16]. As zero-dimensional carbon nanomaterials, CDs have attracted considerable research interest since their discovery owing to their distinctive characteristics [17,18]. With particle sizes typically below 10 nm, CDs exhibit tunable fluorescence, high photostability, good biocompatibility, and low toxicity. Their surfaces are rich in functional groups, which promote effective integration with polymer networks. Moreover, CDs can be synthesized through environmentally benign and facile routes [19,20]. These attributes collectively establish CDs as promising functional components for developing high-performance sensing films.

Nevertheless, several challenges persist in the development of CD-based pH-sensing films. Current research efforts have predominantly emphasized their fluorescence-based sensing capabilities, with limited attention given to the synergistic integration of their fluorescent and electrochemical properties. Furthermore, a systematic understanding of how CDs influence the microstructure, mechanical strength, electrical characteristics, and pH-responsive behavior of starch-based films—particularly those fabricated from low-cost and abundant starch sources—remains insufficient. Clarifying the interfacial interactions between CDs and starch molecules, as well as the underlying mechanism for the enhancement of sensing performance, is also essential.

**Figure 1:**
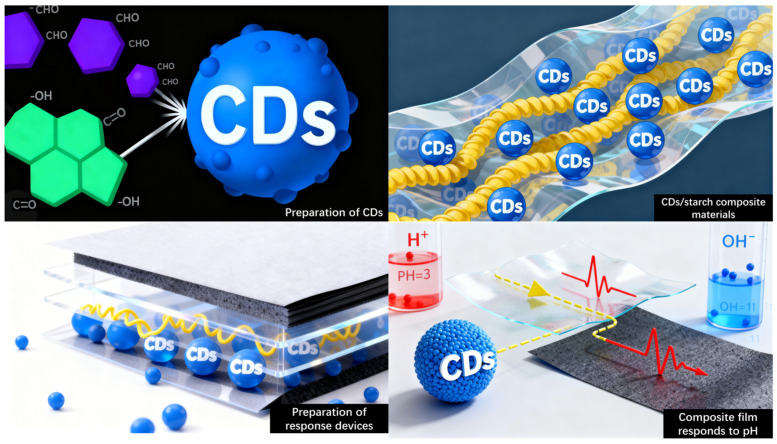
Schematic diagram of pH response of carbon quantum dot starch composite film.

In this context, CDs were synthesized from ellagic acid and phthalaldehyde as carbon precursors and incorporated into amylopectin (CA) and whole-component starch (CS) matrices to fabricate a series of starch-based composite films embedded with CDs. The influence of CDs on the microstructure, mechanical properties, and electrochemical behavior of the films was systematically investigated. The interfacial interaction mechanism between CDs and starch molecules was elucidated using X-ray photoelectron spectroscopy (XPS), among other characterization techniques. The current response of the composite films to buffer solutions with varying pH values was evaluated to assess their performance as electrochemical sensors, leading to the establishment of a robust quantitative pH–current correlation model (Figure 1). Furthermore, the practical applicability of the film was demonstrated through a simulated juice freshness detection experiment, highlighting its potential as a high-performance and environmentally benign material for intelligent food packaging. This study offers a novel strategy for advancing functional sensing platforms in sustainable packaging applications.

## 2. Results and Discussion

### 2.1. Structure and Fluorescence of Ellagic Acid-Based Carbon Dots

**Figure 2:**
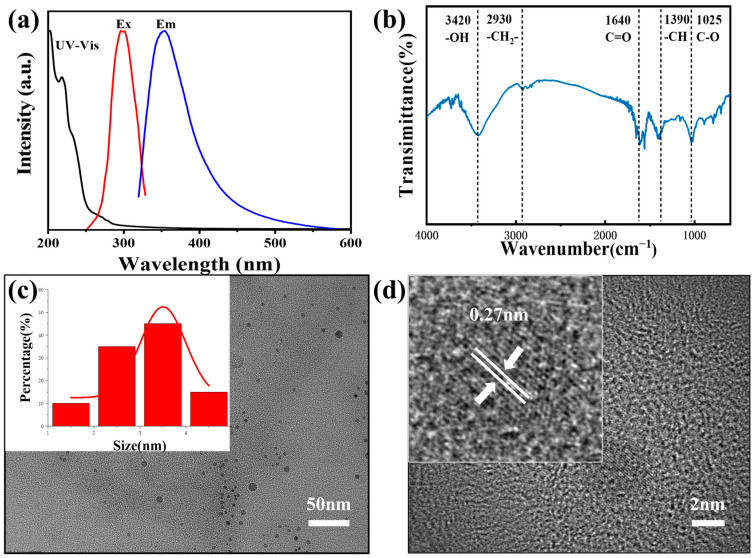
Basic characterization of carbon dots (CDs). (**a**) Fluorescence excitation and emission spectra of CDs; (**b**) FTIR spectrum of CDs; (**c**) Size distribution histogram of CDs; (**d**) HRTEM image showing lattice fringes of CDs.

Carbon dots (CDs) were synthesized from ellagic acid as the carbon source. The UV-Vis absorption spectrum of the CDs displays an intense peak at ~220 nm, which is commonly assigned to the π–π* transition of aromatic C=C bonds in sp^2^-hybridized carbon domains. A prominent tailing feature is observed in the longer wavelength region from ~350 nm to ~550 nm (Figure 2a). This broad, featureless absorption profile strongly suggests an amorphous carbon structure, as opposed to a highly graphitized crystalline core. The presence of abundant oxygen-containing functional groups (e.g., C=O, –OH), as confirmed by FTIR (Figure 2b), gives rise to surface states with a continuous energy distribution, contributing to the broadband absorption spanning the UV to visible region. The absorption tail in the visible range is typically associated with n–π* transitions of C=O bonds and defect-related surface states. Fluorescence spectra reveal a pronounced excitation-dependent emission behavior. As the excitation wavelength increases from 300 nm to 355 nm (Figure 2a), the emission maximum exhibits a systematic redshift from ~300 nm to ~355 nm. Combined with the featureless absorption profile, this marked excitation-dependent fluorescence further supports a surface state-dominated luminescence mechanism. High-resolution transmission electron microscopy (HRTEM) images and corresponding size distribution analysis confirm that the as-synthesized CDs are highly dispersed, quasi-spherical nanoparticles with a narrow size distribution and an average diameter of ~3.2 nm (Figure 2c). Lattice fringes with a spacing of 0.27 nm are clearly resolved (Figure 2d), indicating the presence of a graphitic-like structure within the amorphous carbon framework.

### 2.2. Mechanical Properties of CDs/Starch Composite Films

**Figure 3:**
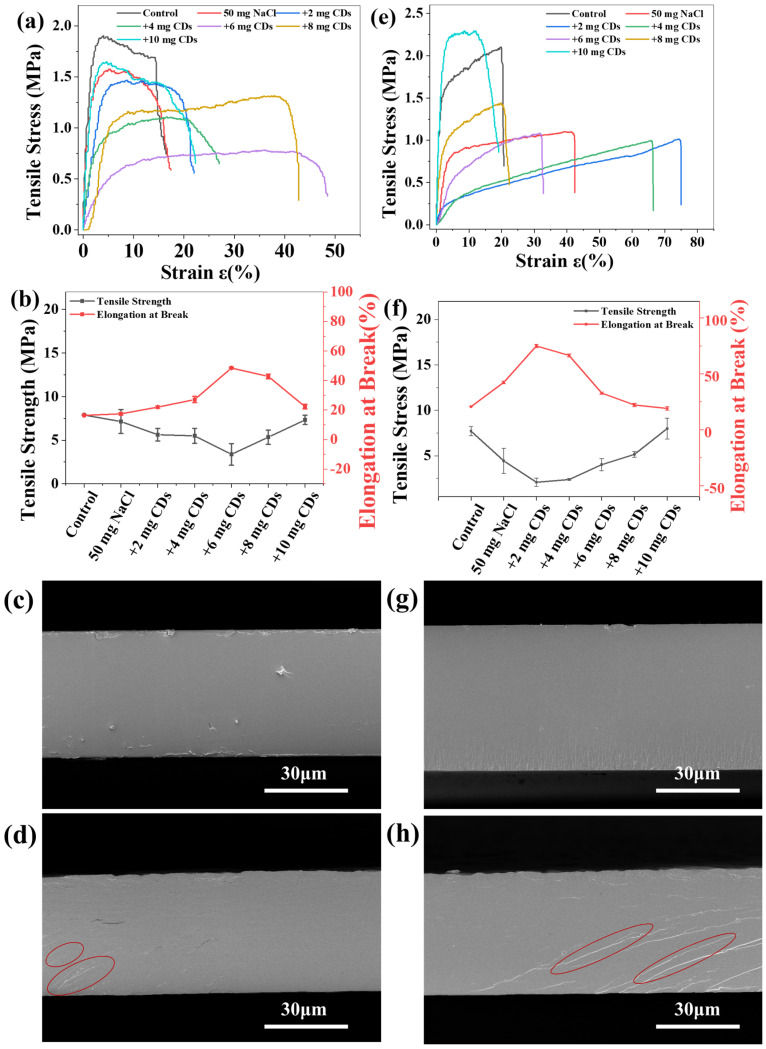
(**a**) Stress–strain curves of CA/CDs composite films; (**b**) Mechanical property trends of CA/CDs composite films; (**c**) SEM image of unmodified CA film; (**d**) SEM image of CA/CDs-10 mg composite film; (**e**) stress–strain curves of CS/CDs composite films; (**f**) Mechanical property trends of CS/CDs composite films; (**g**) SEM image of unmodified CS film; (**h**) SEM image of CS/CDs-10 mg composite film.

CDs/starch composite films were fabricated via a co-precipitation approach. Incorporation of CDs led to a reduction in the yield strength of both amylopectin (CA) and whole-component starch (CS), while the elongation at break initially increased with CD content. The maximum elongation reached 48.1% for CA/CDs and 75.3% for CS/CDs. Beyond a certain loading, however, further addition of CDs resulted in decreased elongation and a corresponding increase in yield strength (Figure 3a,c). These findings indicate that CDs act as a flexible filler, enhancing film toughness up to an optimal concentration. Analysis of the stress–strain behavior suggests that CDs facilitate dislocation motion and increase dislocation storage capacity, thereby improving deformation homogeneity and reducing overall rigidity. To further elucidate the microstructural role of CDs, cryo-fractured cross-sections of the films (CA, CA/CDs-6, CS, CS/CDs-2) were examined. Unmodified starch films exhibited relatively smooth fracture surfaces (Figure 3c,g), whereas CDs-modified films showed characteristic 45° shear bands, indicative of ductile fracture (Figure 3d,h). This morphological transition aligns well with the enhanced elongation observed in mechanical tests.

### 2.3. Crystalline Structure of CDs/Starch Composite Films

**Figure 4:**
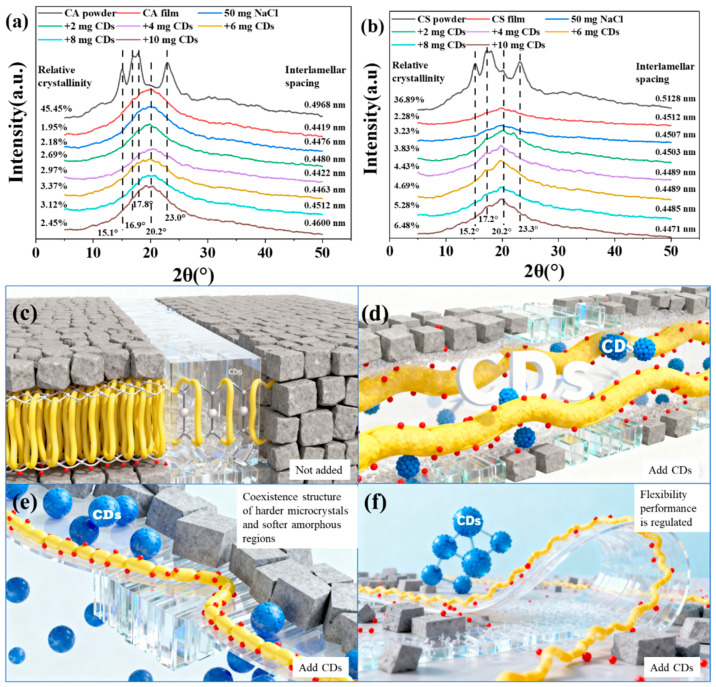
(**a**) XRD patterns of CA/CDs films; (**b**) XRD patterns of CS/CDs films; (**c**) Dense packing of molecular chains in pristine starch; (**d**) Intercalation of CDs between starch chains increasing amorphous chain mobility; (**e**) Formation of a soft-hard coexistence structure with plasticized, deformable amorphous regions and rigid crystalline domains; (**f**) Macroscopically tunable flexibility and toughness of the starch material.

Strain analysis (Figure 3) revealed that the incorporation of CDs reduced the yield strength and enhanced the flexibility of the starch composite films. This phenomenon is attributed to the ability of CDs to lower the resistance to dislocation motion and improve microstructural homogeneity. XRD analysis was performed to further investigate the structural evolution. The native amylopectin and whole-component starch exhibited crystallinities of 45.45% and 36.89%, respectively (Figure 4a,b). In contrast, the unmodified starch films showed significantly reduced crystallinity (CA film: 1.95%; CS film: 2.28%). The CDs-modified starch films displayed only a broad amorphous halo centered around 2θ = 20°, indicating a predominantly amorphous structure (Figure 4a,b). Notably, the introduction of CDs moderately increased the crystallinity of the modified starch films compared to their unmodified counterparts, suggesting a subtle nucleation effect.

The apparent contradiction between increased crystallinity and reduced yield strength can be explained by the dual role of CDs in the starch matrix. As nucleating agents, CDs promote the formation of localized ordered domains, leading to higher crystallinity. Simultaneously, a plasticizing effect predominantly occurs within the amorphous regions, enhancing their softness and deformability (Figure 4c–f). This is attributed to the abundant hydrophilic groups on the CD surfaces (Figure 3b), which endow them with strong water-retention capability. During film preparation and storage, CD adsorbs water from the ambient material. Subsequently, the re-cooling and orientation of molecular chains during composite film formation can lead to the immobilization of this water on the starch surface. These confined water molecules act as internal plasticizers under stress. Furthermore, CDs intercalate between starch molecular chains, disrupting intermolecular hydrogen bonding and thereby increasing chain mobility. This is a manifestation of the nanoscale plasticizing effect. Consequently, the material exhibits a coexistence of “stiffer microcrystalline regions” and “softer amorphous domains,” enabling tunable flexibility in starch-based films through CD incorporation.

### 2.4. Hydrophilicity and Thermal Stability of CDs/Starch Composite Films

**Figure 5:**
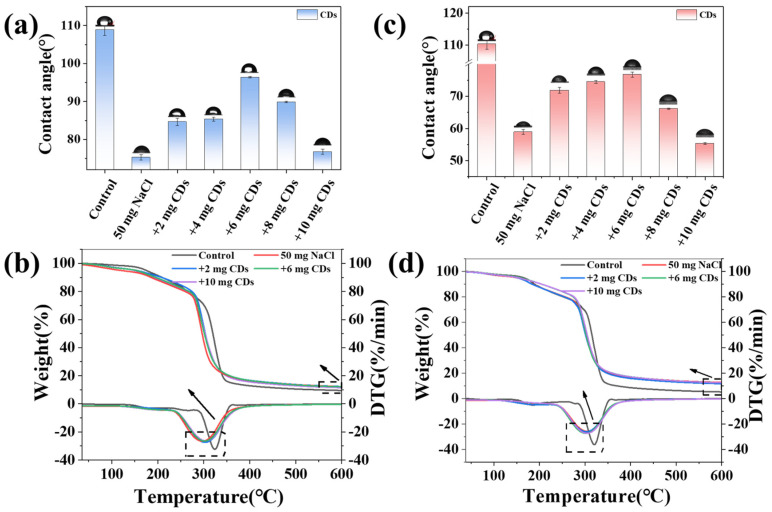
(**a**) Contact angle measurements of the CA/CDs composite film; (**b**) Thermogravimetric analysis (TGA) of the CA/CDs composite film; (**c**) Contact angle measurements of the CS/CDs composite film; (**d**) Thermogravimetric analysis (TGA) of the CS/CDs composite film.

Based on the mechanical performance and crystallinity of the composite films, it was hypothesized that CD incorporation induces a nanoscale plasticizing effect. The formation of hydrogen bonds between CDs and starch disrupts the original continuous structure of the starch matrix. This structural alteration consequently leads to a decrease in tensile strength while simultaneously enhancing the toughness of the composite film. Although CDs also function as nucleating agents to promote crystallinity, this effect is overshadowed by the dominant plasticizing behavior. To validate this hypothesis, differences in water adsorption between composite and pure starch films were evaluated to determine whether CD-modified materials retain higher moisture content. After CDs incorporation, the contact angles of both CA/CDs and CS/CDs films decreased markedly (Figure 5a,c), indicating enhanced hydrophilicity. Thermogravimetric analysis further revealed the influence of CDs on the thermal degradation behavior of the films. The maximum weight-loss temperature of the CD-composite films was lower than that of pure starch films (Figure 5b,d), confirming reduced thermal stability due to CD incorporation. Together, these results corroborate the increased hydrophilicity of the composite films, which accounts for the observed nanoscale plasticizing effect.

### 2.5. Chemical Structure Analysis of CDs/Starch Composite Films

**Figure 6:**
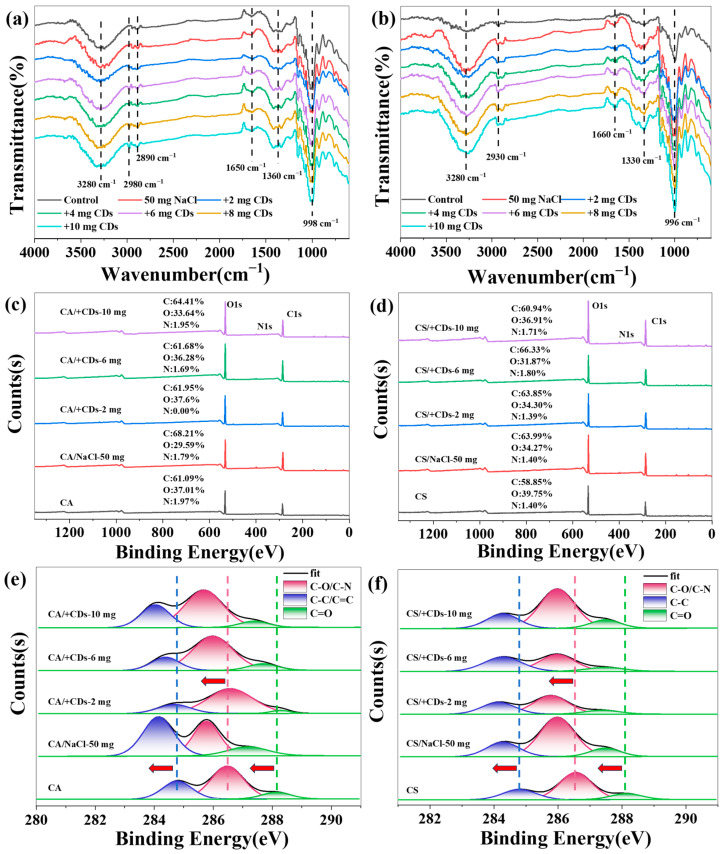
(**a**) FTIR spectrum of CA/CD film; (**b**) FTIR spectrum of CS/CD film; (**c**) Elemental map of CA/CDs film; (**d**) Elemental map of CS/CDs film; (**e**) C 1s XPS spectrum of CA/CD film; (**f**) C 1s XPS spectrum of CS/CDs film.

Fourier transform infrared (FTIR) analysis revealed that amylopectin and whole-component starch exhibit similar characteristic peaks, and the incorporation of CDs did not significantly alter the characteristic functional groups of the starch films (Figure 6a,b). The absorption band near 1650 cm^−1^ is attributed to the H–O–H. Furthermore, its consistent presence and enhanced intensity in the composite films compared to the pure starch film suggest that the inclusion of CDs increases the overall hydrophilicity of the film matrix, thereby facilitating water adsorption [21].

X-ray photoelectron spectroscopy (XPS) analysis of the C 1s spectra in starch-based/CDs composite films identified characteristic peaks at approximately 284.5 eV (C–C/C=C), 286.0 eV (C–O in starch and CDs chains), and 287.6 eV (C=O). Notably, all these peaks exhibited a consistent shift toward lower binding energies in both CA/CDs and CS/CDs composites (Figure 6e,f), indicating substantial electron interaction between the starch matrix and CDs. This shift can be attributed to hydrogen bonding between oxygen atoms in CDs and hydroxyl groups in starch, which promotes delocalization of electron density. The conjugated aromatic core of CDs constitutes an extensive π–electron system. Upon hydrogen bonding with starch, electron density from this electron-rich system partially redistributes toward carbon atoms in starch molecules, leading to increased electron density on starch and a corresponding negative binding energy shift. This hydrogen bond-mediated electronic influence is not confined to specific bonds but affects the entire carbon backbone environment, demonstrating that CDs engage in broad, integrative interactions with starch molecules rather than localized reactions with particular functional groups.

**Figure 7:**
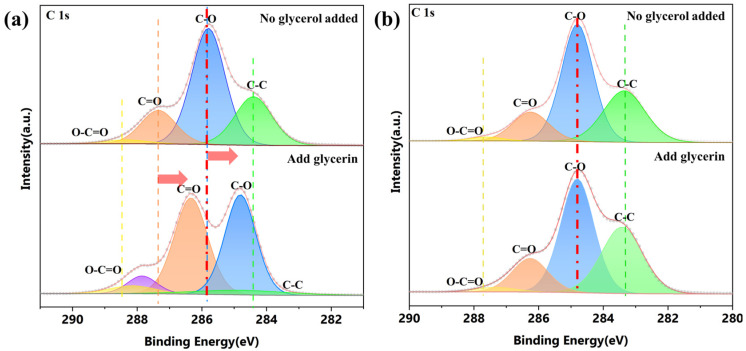
(**a**) C 1s spectra of the CA film before and after glycerol addition; (**b**) C 1s spectra of the CS film before and after glycerol addition.

In order to verify the main factors that can be reduced by the combination, we conducted XPS analysis on CS film and CA starch film before and after glycerol addition. The results showed that the C–O bond position in the CS membrane remained unchanged after adding glycerol (Figure 7a). In contrast, after glycerol treatment, all characteristic peaks in the CA membrane shifted towards lower binding energies (Figure 7b). These findings indicate that the XPS displacement observed in CS/CDs composite films is entirely caused by the incorporation of CDs, while the displacement in CA/CDs composite films is caused by the combined action of glycerol and CDs.

### 2.6. Electrical Properties of CDs/Starch Composite Films

**Figure 8:**
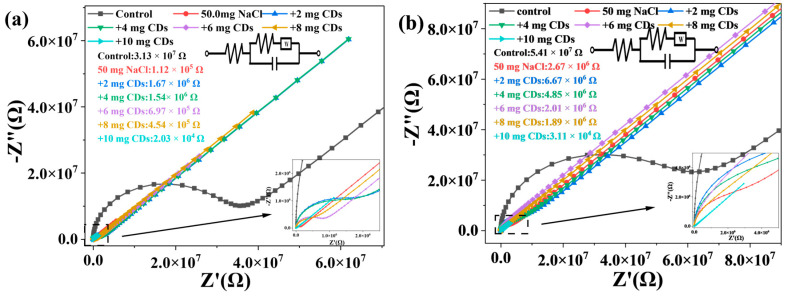
(**a**) EIS of CA/CDs film; (**b**) EIS of CA/CDs film.

The electrical impedance of pure CA and CS films was measured at 3.13 × 10^7^ Ω and 5.41 × 10^7^ Ω, respectively. The incorporation of CDs progressively reduced the impedance of the composite films, with the lowest value achieved at a CDs loading of 10 mg (Figure 8a,b). This behavior suggests that CDs form conductive pathways within the starch matrix, and an optimal loading level exists beyond which no further enhancement in conductivity is observed.

### 2.7. Fluorescence Properties of CDs/Starch Composite Films

**Figure 9:**
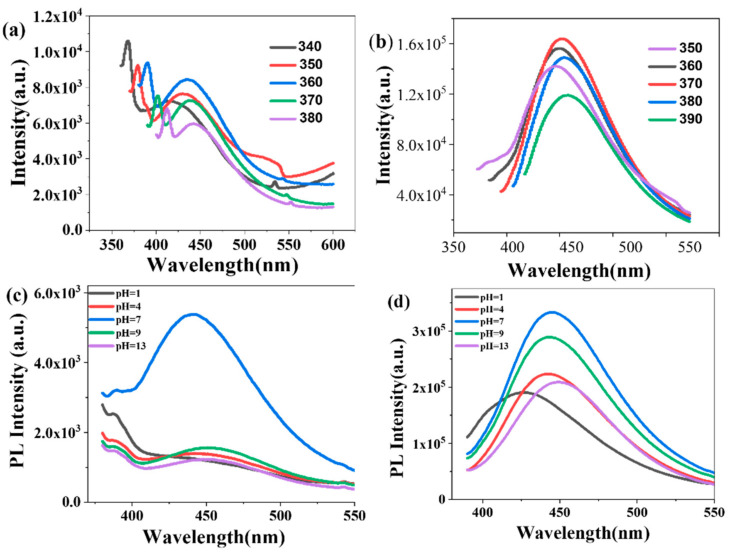
(**a**) Fluorescence excitation spectrum of the CA/CDs film; (**b**) Fluorescence excitation spectrum of the CS/CDs film; (**c**) Fluorescence emission spectrum of CA/CDs; (**d**) Fluorescence emission spectrum of CS/CDs.

The composite film with the lowest electrical impedance (10 mg CDs loading) was selected to investigate the fluorescence properties of CDs. The fluorescence emission spectrum of the CA/CDs-10 film exhibited a notable red shift in the emission maximum as the excitation wavelength increased from 360 nm to 400 nm (Figure 9a). Maximum fluorescence intensity was observed at an excitation wavelength of 360 nm, with a corresponding emission peak at 436 nm. Based on this, an excitation wavelength of 360 nm was employed for pH-dependent measurements. The CA/CDs-10 film demonstrated the strongest fluorescence intensity at pH 7, with a noticeable decrease under both acidic and alkaline conditions (Figure 9c). This fluorescence quenching is likely attributable to protonation-deprotonation processes of functional groups on the CDs, facilitating photoinduced electron transfer and subsequent quenching. Similarly, the CS/CDs-10 film showed maximum fluorescence intensity at an excitation wavelength of 370 nm (Figure 9b), with the highest intensity also occurring at pH 7 (Figure 9d). The consistent pH-dependent fluorescence behavior observed in both types of composite films confirms their potential for use in acid-base sensing in liquid environments.

### 2.8. pH-Responsive Behavior and Sensing Performance of CDs/Starch Composite Films

**Figure 10:**
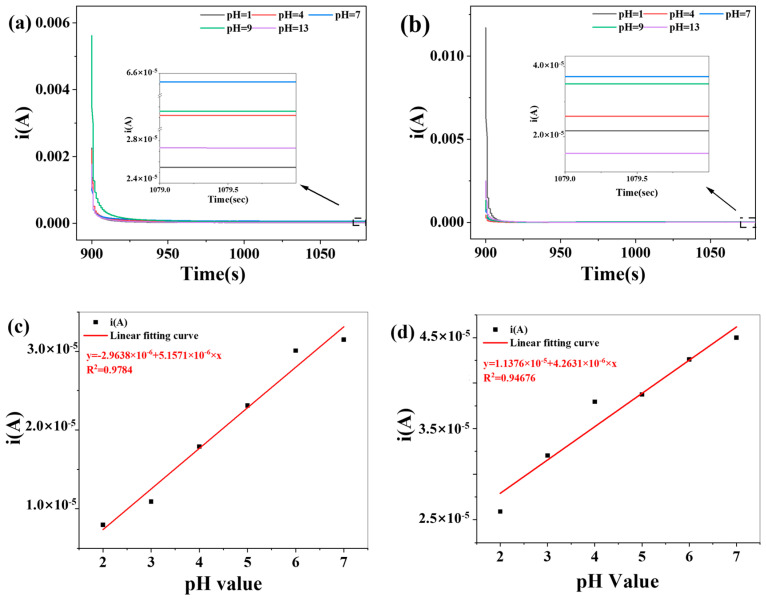
(**a**) Chronoamperometric curves of the CA/CDs composite film at various pH levels; (**b**) Chronoamperometric curves of the CS/CDs composite film at various pH levels; (**c**) Calibration curve of current vs. pH for the CA/CDs composite film; (**d**) Calibration curve of current vs. pH for the CS/CDs composite film.

The electrochemical pH response of the CA/CDs-10 and CS/CDs-10 composite films was evaluated through chronoamperometric measurements across different pH environments. The steady-state current ranged from 2.62 × 10^−5^ A to 6.35 × 10^−5^ A for CA/CDs-10 and from 1.54 × 10^−5^ A to 3.73 × 10^−5^ A for CS/CDs-10 (Figure 10a,b). Both films exhibited a consistent current–pH dependence, with the maximum current observed at pH 7 and a gradual decrease under more acidic or alkaline conditions. Within the acidic range (pH 2–7), the steady-state current showed a positive correlation with increasing pH. The stable current responses of the starch-based/CDs-10 composite films were analyzed and demonstrated a positive correlation with environmental pH. The pH-current relationship for the CA/CDs-10 composite film was fitted to a linear equation with a high coefficient of determination (*R*^2^ = 0.9784; Figure 10c). Similarly, the CS/CDs-10 composite film also exhibited a strong linear response, yielding an *R*^2^ value of 0.9468 (Figure 10d). With both determination coefficients (*R*^2^) exceeding 0.9, a statistically significant linear correlation between pH and current is confirmed. The pronounced linear relationship within the pH 2–7 range demonstrates the potential of these starch-based/CDs films in food pH detection and real-time monitoring applications.

### 2.9. Application of CDs/Starch Composite Films in Juice Freshness Detection

**Figure 11:**
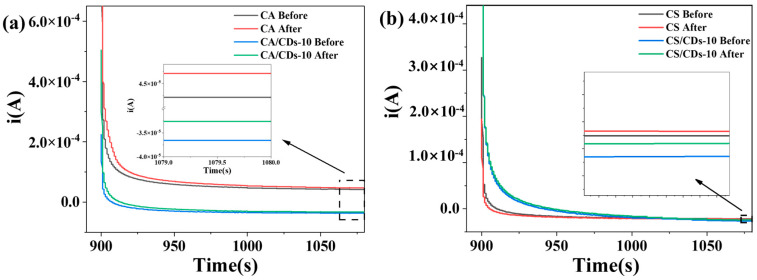
(**a**) Chronoamperometry curves of CA/CDs film in liquid foods; (**b**) Chronoamperometry curves of CS/CDs film in liquid foods.

To evaluate the practical applicability of the composite films in beverage quality monitoring, fresh (pH 3.38) and spoiled (pH 2.68) orange juices were analyzed using self-supported sensor chips based on the starch-based/CDs films. Chronoamperometric measurements revealed distinguishable current responses between the two juice states. For the CA-based system, the pristine CA film showed current magnitudes of 4.21 × 10^−5^ A and 4.69 × 10^−5^ A for fresh and spoiled juice, respectively, corresponding to an increase of 4.8 × 10^−6^ A upon spoilage. The CA/CDs-10 composite film exhibited currents of 3.6 × 10^−5^ A (fresh) and 3.4 × 10^−5^ A (spoiled), with a difference of 2.0 × 10^−6^ A (Figure 11a). In the CS-based system, the pristine CS film recorded currents of 2.18 × 10^−5^ A (fresh) and 2.23 × 10^−5^ A (spoiled), reflecting a spoilage-induced increase of 5 × 10^−7^ A. The CS/CDs-10 composite exhibited currents of 2.76 × 10^−5^ A and 2.46 × 10^−5^ A for fresh and spoiled juice, respectively, differing by 3 × 10^−6^ A (Figure 11b). The consistent increase in current magnitude with juice spoilage across both film types demonstrates the capability of these materials to detect quality changes through pH-dependent electrochemical signals. These results confirm the feasibility of using such starch-based/CDs composite films for rapid, non-destructive quality assessment of liquid foods.

## 3. Materials and Methods

### 3.1. Experimental Materials

Amylopectin (CA, Analytical Reagent) and Complete Starch (CS, Analytical Reagent) were purchased from Aladdin Chemical Company (Shanghai, China). Other chemical reagents, including ellagic acid (Analytical Reagent) and o-Phthalaldehyde (Analytical Reagent), were also obtained from Aladdin. Sodium hydroxide (NaOH, Analytical Reagent) was supplied by Sinopharm Chemical Reagent Co., Ltd. (Beijing, China).

### 3.2. Preparation of Ellagic Acid Carbon Dots

CDs were synthesized via a hydrothermal method. Briefly, 0.5 g of ellagic acid, 0.5 g of phthalaldehyde, and 10 mL of anhydrous ethanol were placed in a Teflon-lined autoclave. The autoclave was heated at 180 °C for 14 h in a muffle furnace. After cooling naturally to room temperature, the resulting suspension containing carbon dots was collected. The crude product was purified by silica gel column chromatography using anhydrous ethanol as the eluent. Finally, the purified CDs were obtained after solvent removal under vacuum drying.

### 3.3. Preparation of Starch Film

The starch-based composite films were fabricated via a solution casting method. Specifically, 4 g of starch (either CA or CS), 1.4 g of glycerol (accounting for 35 wt% of the starch), and 96 mL of ultrapure water were added into a three-neck flask. The mixture was stirred at room temperature for 30 min, then transferred to a constant-temperature water bath maintained at 90 °C and stirred for another 50 min. Thereafter, 10 mg of NaCl and varying amounts of CDs (0, 2, 4, 6, 8, and 10 mg) were sequentially added. After thorough mixing, the resulting starch/CDs film-forming solution was labeled as starch/CDs-x, where “x” denotes the mass (in mg) of CDs incorporated. For instance, CA/CDs-10 refers to the amylopectin-based film with 10 mg of CDs. Each film-forming solution was cast into a polytetrafluoroethylene Petri dish (15 cm in diameter) and dried at 45 °C for 6 h to obtain the corresponding film. The naming convention for the final films is consistent with that of the precursor solutions.

### 3.4. Methods

The microstructure and particle size distribution of the CDs were characterized using a high-resolution transmission electron microscope (HR-TEM, TECNAI G2 F20, Thermo Fisher Scientific, Waltham, MA, USA). The crystal structure of the composite films was examined by X-ray diffraction (XRD, Max220, Hitachi, Tokyo, Japan). Fourier transform infrared spectroscopy (FTIR, Nicolet iS50, Thermo Fisher Scientific, USA) was employed to identify the chemical functional groups. X-ray photoelectron spectroscopy (XPS, K-Alpha, Thermo Fisher Scientific, USA) was used to analyze the interfacial chemical interactions. Tensile properties, including tensile strength and elongation at break, were measured with a universal testing machine (Instron 5967, Instron, Norwood, MA, USA) following the ASTM D882 standard. Specimens were cut into strips (10 mm × 50 mm) and tested at a crosshead speed of 10 mm/min. The surface wettability was evaluated by measuring the water contact angle using a goniometer (OCA20, DataPhysics Instruments GmbH, Filderstadt, Germany). Thermal stability was assessed by thermogravimetric analysis (TGA, Q50, TA Instruments, New Castle, DE, USA). Electrochemical impedance spectroscopy (EIS) was performed on an electrochemical workstation (CHI760E, CH Instruments, Inc., Shanghai, China). Fluorescence excitation and emission spectra were acquired on a fluorescence spectrophotometer (F-7000, Hitachi High-Tech Corporation, Tokyo, Japan). The fluorescence properties were investigated by recording emission spectra under different excitation wavelengths.

## 4. Conclusions

This work presents the synthesis of carbon dots (CDs) with excellent fluorescence properties via a one-step hydrothermal method, and their incorporation into amylopectin (CA) and whole-component starch (CS) matrices to fabricate a series of starch-based composite films. Comprehensive characterization confirmed that CDs interact with starch molecules through hydrogen bonding, effectively modifying the microcrystalline structure and significantly enhancing film flexibility. The dual functionality of CDs—acting as both a plasticizer and a nucleating agent—synergistically modulated the film microstructure, resulting in superior deformability while retaining a partially ordered framework. Electrochemical impedance and fluorescence analyses demonstrated that CD incorporation markedly reduced film impedance and endowed the composites with pronounced pH-responsive fluorescence. A strong linear correlation (*R*^2^ > 0.94) was established between current response and pH in the range of 2–7, confirming reliable acid-base sensing performance. In simulated juice spoilage tests, the CDs-modified films effectively discerned current signal variations corresponding to pH changes. Overall, this study elucidates the multi-functional role of CDs in modifying starch-based composites and introduces a highly sensitive, rapid-response pH-sensing film, establishing a practical material platform and methodological approach for non-destructive food quality monitoring.

## Data Availability

The data presented in this study are available on request from the corresponding author.
